# Clear cell unicystic ameloblastoma

**DOI:** 10.4103/0973-029X.80013

**Published:** 2011

**Authors:** MB Radhika, Lalita J Thambiah, K Paremala, M Sudhakara

**Affiliations:** *Department of Oral Pathology, Krishnadevaraya College of Dental Sciences, MVIT Campus, Near Yelahanka, Hunusamaranahalli, Bangalore, India*

**Keywords:** Ameloblastoma, clear cell, unicystic variant

## Abstract

Clear cell differentiation in unicystic ameloblastoma with inclusion of many other histologic variants in the same tumor is a very rare occurrence. Here, we report a case of a well-circumscribed large mandibular swelling in a 22 - year old female. The lesion was histopathologically diagnosed as unicystic ameloblastoma which showed multiple histologic patterns and clear cell differentiation. The tumor was treated with surgical enucleation and chemical cauterization. A follow up of 20 months has shown no recurrence after initial surgery.

## INTRODUCTION

Ameloblastoma is the most common odontogenic neoplasm. Churchill is credited with the first use of the term ameloblastoma in 1934.[1] A thorough description of an ameloblastoma was given by Falkson in 1879, and since then, thousands of reports on ameloblastoma have been published.[1] More than 80% of all ameloblastomas are solid or multicystic variants, with unicystic ameloblastoma being an important clinicopathologic form of ameloblastoma and occupying the other 20% of the cases along with peripheral ameloblastoma. Robinson and Martinez in 1977 were the first to draw a clear distinction between unicystic ameloblastoma and to call for recognition of the entity.[1]

Histologically, various patterns are seen in ameloblastoma, with follicular and plexiform variants being more common. Less common histopathologic patterns include acanthamatous, granular cell, basal cell type, desmoplastic and keratoacanthoma types.[1]

On the other hand, the unicystic ameloblastoma histologically generally shows plexiform and follicular pattern along with a cystic lining of ameloblastic epithelium. The histologic criteria for the diagnosis of unicystic ameloblastoma as described by Vickers and Gorlin includes a cyst lined by ameloblastic epithelium with a tall columnar basal layer, subnuclear vacuoles, reverse polarity of hyperchromatic nucleus and a thin layer of edematous, degenerating stellate reticulum like cells on the surface.[13] The mural extension into the cystic wall is a frequently seen feature. Sometimes, the lesion also shows intraluminal growth of hyperplastic, often inflamed epithelium.[3] The unicystic ameloblastoma is set to be less aggressive clinically than the other variants and has a better prognosis.[2,3]

Clear cell odontogenic tumor is an uncommon or unusual neoplasm of the jaw. According to Lewis *et al*., the first case of clear cell neoplasm of odontogenic origin was attributed to Hansen *et al*,[4] The term “clear cell ameloblastoma” (CCA) was designated by Waldron *et al*. in 1995.[5] Since then, only a few cases have been reported.[5,6] CCA has a clinically aggressive course, and most of the times, it shows recurrence and metastasis which indicates the need to consider this neoplasm as a low-grade odontogenic carcinoma.[5,6] Histologically, most of the CCAs show biphasic pattern.[6]

Here, we report a rare case of unicystic ameloblastoma showing histopathologically clear cell differentiation and a plethora of other histologic patterns of ameloblastoma like follicular, plexiform, basaloid, acathomatous differentiation with both intraluminal and mural growth.

## CASE REPORT

A 22-year old systemically healthy Indian lady reported to an Oral and Maxillofacial surgeon with a chief complaint of swelling in the right lower jaw since 3 months. The swelling was asymptomatic and gradually increased to the present size.

**Figure 1:**
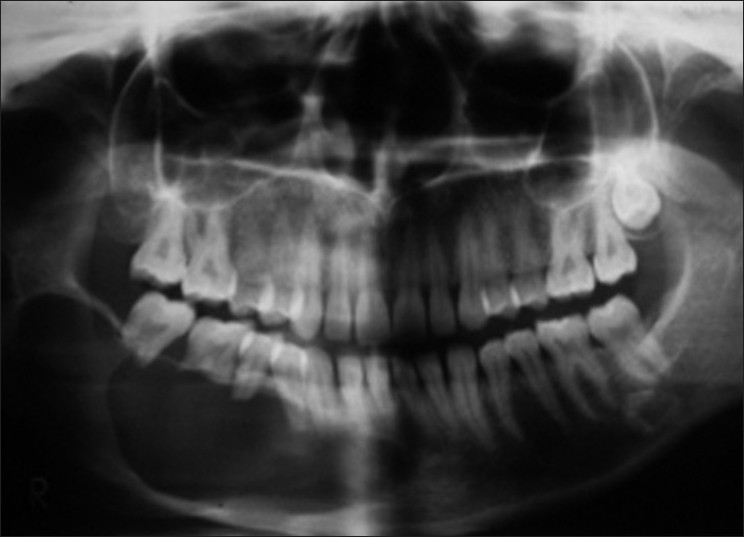
A large, unilocular radiolucency involving the right side of the mandible. Well-demarcated lesion involving 41–47 region. Destruction of buccal plate and lingual plate is seen. Extensive root resorption of 46 and 47 is seen. Lower border of mandible is intact

Extraoral examination revealed a bony hard swelling measuring about 5.0 × 4.0 cm in dimension. The lesion extended from the corner of the mouth to the posterior part of the body of the mandible on the right side. The overlying skin and mucous membrane were stretched but intact. On intraoral examination, the swelling showed obliteration of the right lower vestibule extending from 42 to 47 region, with extension on the lingual aspect. The teeth showed grade I mobility in relation to 42 - 44 and grade II mobility in relation to 45 - 47. The orthopantamogram showed a single, large, unilocular radiolucent lesion extending from 42 region to right ramus of mandible with a thin rim of well-demarcated radioopacity. Loss of buccal plate was more extensive than the lingual plate. The involved teeth (42 - 47) showed varying degrees of extensive root resorption with loss of interdental bone giving a floating teeth appearance [Figure 1]. Inferior mandibular canal showed a wash rope appearance. The lesion was surgically enucleated under general anesthesia and chemical cauterization was done with Carnoy’s solution. The surgical site was packed with 5% betadine pack. The patient was kept under close postoperative observation. The healing was uneventful and the patient was given an acrylic plate with clasps (artificial prosthesis). After a regular follow-up for 20 months, there was no evidence of any clinical recurrence.

## HISTOPATHOLOGY

The hematoxylin and eosin (H and E) stained sections showed a cystic lining made of spongiotic odontogenic epithelium of varied thickness ranging from few cell layers to multiple layers. Most of the areas showed basal cells made up of ameloblast - like cells with palisading nucleus exhibiting reverse polarity. Subepithelially, hyalinization was predominantly seen, while some areas showed chronic inflammation.

**Figure 2:**
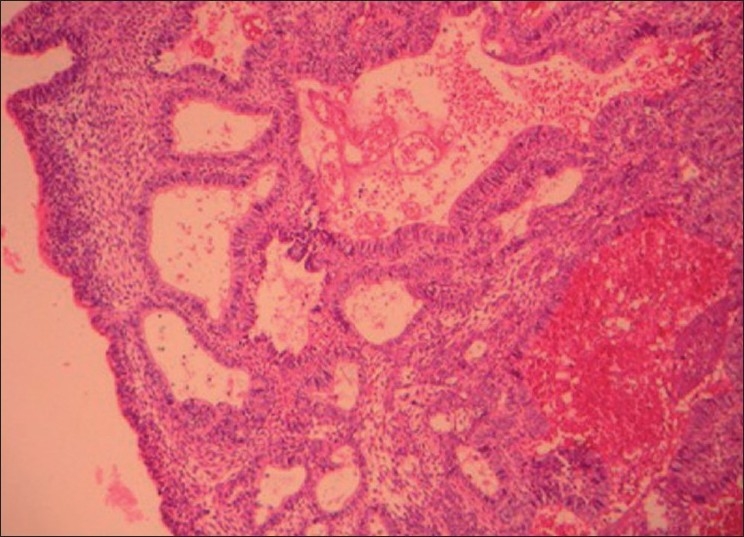
Photomicrograph showing intraluminar proliferation of tumor cells made of palisading chords of plexiform pattern and sheets of basaloid cells. Few stromal cysts are seen along with capillaries (H and E, 100×)

**Figure 3:**
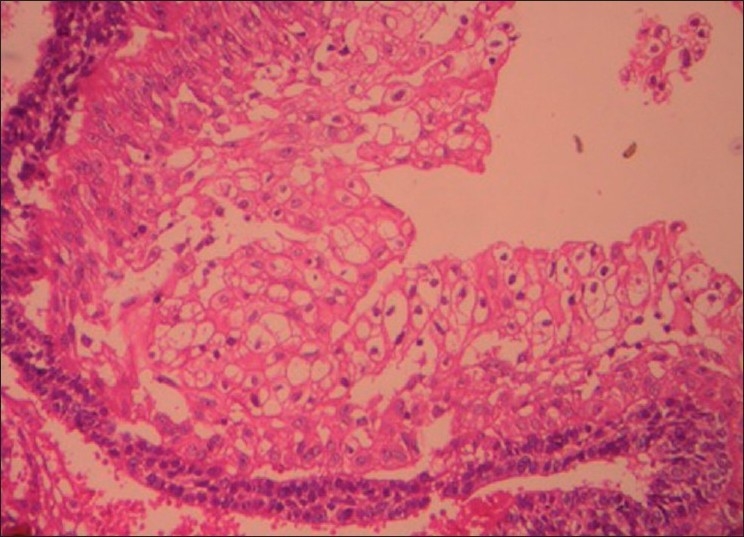
Photomicrograph showing follicles with groups of clear cells of varying sizes with clear cytoplasm and single centrally placed nucleus (H and E, 200×)

**Figure 4:**
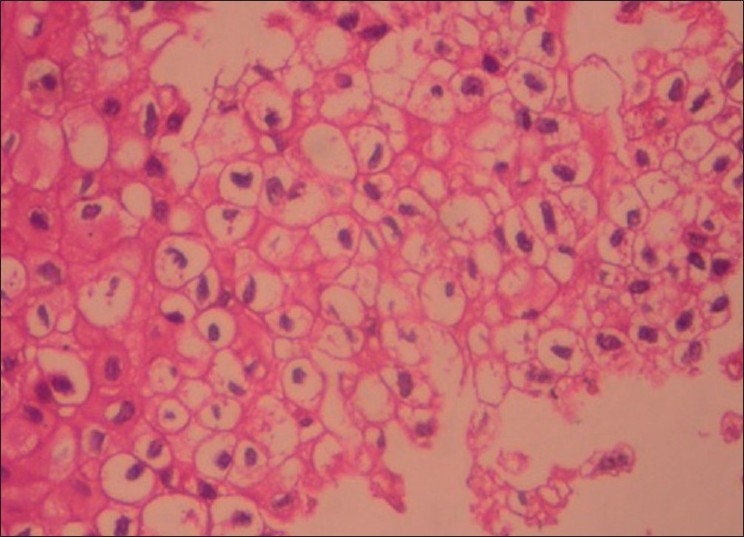
Photomicrograph showing clear cells seen as oval or round cells with clear cytoplasm and prominent centrally placed hyperchromatic nucleus (H and E, 400×)

The cystic lining at numerous areas showed intraluminal extensions of nodules that were filled with densely packed plexiform strands and large solid follicles of basaloid cells [Figure 2]. Rich capillary spaces were seen in the vicinity of the epithelium of the hyperplastic intraluminal growth. The basaloid follicles showed a border of ameloblast-like cells and a core of compactly packed small cells with prominent large nucleus. A large number of other follicles of the intraluminal growth contained clear cells that were numerous in number, small to large in size with clear cytoplasm and single pyknotic nucleus [Figures 3 and 4]. Most of the clear cells were seen in the area of stellate reticulum-like cells and were admixed with odontogenic epithelial cells forming keratin.

**Figure 5:**
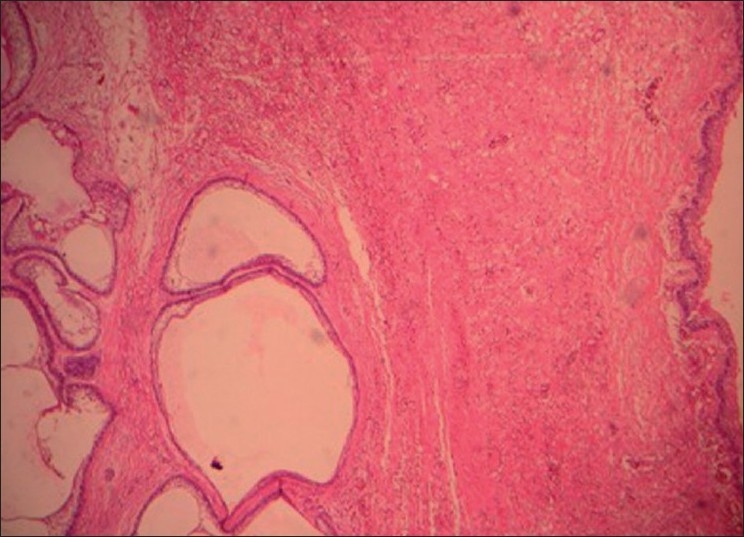
Photomicrograph showing a cystic epithelium on the right side. Spongiotic cystic epithelium satisfying Vicker and Gorlin criteria. On the left are seen small and large ameloblastic follicles as intramural invasion. Few follicles show cystic degeneration and acanthomatous change, all set in a bland stroma (H and E, 100×)

In the connective tissue wall, numerous long, ribbon-like strands of bilaminar ameloblast-like cells were seen forming a plexiform pattern. Numerous follicles were seen typically as in follicular ameloblastoma wherein they showed a single layer of tall columnar ameloblast-like cells with palisading nucleus exhibiting reverse polarity and a core of stellate reticulum-like cells. Some of the follicles showed squamous metaplasia in the stellate reticulum-like area, whereas some showed cystic degeneration [Figure 5]. The connective tissue wall was thick and showed abundant collagen.

Special staining of the tissues with PAS showed positivity in the epithelial cells that are present along with the clear cells and appeared pink in color, whereas the clear cells themselves did not take up the stain and hence appeared negative.

## DISCUSSION

Odontogenic tumors of the jaws with large or predominant clear cell differentiation are rare.[5,6] Presence of clear cells in the odontogenic tumors should not be considered surprising because of their origin from the dental lamina that have clear cell components.[7] In the orofacial region, it is important to make definitive diagnosis for a clear cell neoplasm based on clinical and histopathologic features.[4,8] The odontogenic clear cell tumor should also be differentiated from the other clear cell tumors such as clear cell variant of mucoepidermoid carcinoma, clear cell squamous cell carcinoma, metastatic renal carcinoma, etc.[9] In the present case, a classical picture of unicystic ameloblastoma was seen along with clear cell differentiation with other histopathologic patterns. This made our diagnosis of unicystic ameloblastoma easier.

Clinically, ameloblastomas are generally intraosseous tumors of the jaw bones with male to female ratio being 1:1.3. The commonly affected sites are mandibular molar and ascending ramus regions. The age at the time of diagnosis ranges from 33 to 44 years. In unicystic ameloblastoma, the age is considerably lower and ranges from 19 to 27 years. Radiographically, most of ameloblastomas show multilocularity, whereas unilocular ameloblastomas show a single large unilocular radiolucency. The involved teeth show varying degrees of root resorption.[3]

Clinically and roentgenographically, the present case mimics the typical presentation of unicystic ameloblastoma. The 22-year-old Indian lady showed a bony hard swelling with egg shell crackling. The roentgenograph showed a single large unilocular radiolucency which had destroyed the buccal and the lingual plate considerably. The involved teeth 42-47 showed a good amount of root resorption, with 46 and 47 showing almost complete loss of roots.

On histopathologic examination, the present case showed classical features of unicystic ameloblastoma. The cystic epithelium almost completely lined the cystic lumen and satisfied the Vickers and Gorlin’s criteria. According to classification given by Ackermann **et al**., our case falls in groups 1, 2, and 3 as the feature of intraluminal extension and mural invasion of the cyst wall is seen along with the cystic lining. As the standardization given by Marx and Stern, the present lesion shows both intraluminal ameloblastoma *in situ* and transmural microinvasive ameloblastoma. Other histopathologic variants like follicular with cystic degeneration, plexiform, acanthomatous, and basaloid types were also seen. In addition to the above variants, a good population of clear cells was seen in the follicles at numerous areas in the intraluminal growth. The cells were predominantly large in size with completely clear cytoplasm and a single pyknotic nucleus. The cytoplasmic clearing in clear cells may be because of the presence of cytoplasmic contents like glycogen[2] or due to sparsity of cellular organelles.[10] As glycogen is stored in oral epithelial cells, PAS staining was done wherein most of the clear cells failed to take up the stain suggesting that the sparsity of intracellular organelles might have given the clear appearance rather than enriched substances like glycogen. As the lesion showed a large number of such clear cells, it is considered under the category of clear cell odontogenic tumor (CCOT).

CCOTs are mainly clear cell odontogenic carcinoma (CCOC) and CCA/malignant clear cell ameloblastoma. Reichart and Philipsen believe that CCOC and CCA/malignant clear cell ameloblastoma constitute two separate tumors. More cases studies are needed to reveal if CCOC and CCA are separate entities or variants of a biological and histopathologic spectrum of clear cell carcinomas. The WHO classification of odontogenic tumors recognizes CCOTs as a distinct entity,[6] while waiting for its phylogenetic classification. Because of potentially aggressive behavior and metastasis, Eversole concluded that CCOTs should be classified as carcinomas.[6]

CCAs should be individualized as a histologic variant of ameloblastoma.[5,11] They show unusual histologic biphasic patterns with areas of acceptable ameloblastoma (follicular, basaloid cells, acanthomatous) together with the conspicuous clear cell component in the ameloblastic follicles.[4,5] The presence of clear cell component may represent a sign of dedifferentiation and possibly a malignancy with or without metastases.[5]

Most of the CCOTs show a biphasic histologic pattern with nests and cords of clear cells and areas of ameloblastic differentiation showing nuclear polarization, peripheral palisading, squamous differentiation, and cystic spaces. Sometimes, dystrophic calcifications were seen and were associated with aggressive behavior.[6] Hence, it was proposed not to call these lesions as clear cell ameloblastomas as it misleads about the aggressive behavior of this lesion.[12] Waldron *et al*. suggested the term clear cell ameloblastoma as low-grade odontogenic carcinoma, hence proposed the use of the term clear cell ameloblastic carcinoma.[13,14] Among the various histologic subtypes of ameloblastoma, the granular cell variant is believed to be more aggressive in behavior, whereas unicystic/cystic ameloblastomas exhibit a low rate of recurrence after enucleation/curettage.[15] It is of general consensus that unicystic CCA is the less aggressive intraosseous variant of ameloblastoma.[2] Recurrence rate for unicystic ameloblastoma is 10-15%. In the present case, all the features point toward a unicystic ameloblastoma with intraluminal proliferation showing clear cell differentiation in the follicles and evidence of mural invasion. Other than the mural invasion, the cells did not show any other signs of atypia, mitotic figures or dysplasia. Absence of features of cellular atypia leads to the lesion being called as clear cell ameloblastoma.

The importance of presence of cellular atypia before labeling it as a malignancy is stressed well in a case of clear cell peripheral ameloblastoma and in few other case reports.[15]

There is no evidence of clinical recurrence for 20 months after the initial treatment. In our case, the presence of clear cells in the tumor has not altered its potential biologic behavior. Nonetheless, a long-term follow-up in such cases is a necessity.

## CONClUSION

Clear cell differentiation in some lesions might not necessarily indicate aggressive behavior, especially of the innocuous variants like unicystic and peripheral ameloblastomas. Hence, presence of clear cells in ameloblastomas can be categorized as benign CCAs if there is absence of atypia and dysplastic features. Caution should be exercised before we call it as a carcinoma as the treatment modality for both the lesions varies drastically.
